# Leisure-time physical activity and DNA damage among Japanese workers

**DOI:** 10.1371/journal.pone.0212499

**Published:** 2019-02-15

**Authors:** Ryoko Kawakami, Ikuko Kashino, Hiroshi Kasai, Kazuaki Kawai, Yun-Shan Li, Akiko Nanri, Mitsuru Higuchi, Tetsuya Mizoue

**Affiliations:** 1 Faculty of Sport Sciences, Waseda University, Tokorozawa, Japan; 2 Department of Epidemiology and Prevention, Center for Clinical Sciences, National Center for Global Health and Medicine, Tokyo, Japan; 3 Department of Environmental Oncology, Institute of Industrial Ecological Sciences, University of Occupational and Environmental Health, Fukuoka, Japan; 4 Department of Food and Health Sciences, International College of Arts and Sciences, Fukuoka Women’s University, Fukuoka, Japan; University of Maiduguri College of Medical Sciences, NIGERIA

## Abstract

**Background:**

It remains unclear whether daily physical activity is associated with DNA damage. This cross-sectional study examined the association between leisure-time physical activity and urinary 8-hydroxydeoxyguanosine (8-OH-dG), a biomarker of oxidative DNA damage, or urinary 7-methylguanine (m^7^Gua), a biomarker of methylating DNA damage.

**Methods:**

Participants included 501 workers (294 men and 207 women), aged 20–65 years, from municipal offices in Japan. Urinary 8-OH-dG and m^7^Gua were measured using column-switching HPLC. Physical activity was evaluated using a self-reported questionnaire. The associations between leisure-time physical activity and urinary DNA damage markers were assessed by multiple linear regression analysis, with stratification by occupational physical activity.

**Results:**

After adjusting for covariates, leisure-time physical activity showed a suggestive inverse correlation with urinary 8-OH-dG levels (*P* for trend = 0.06), and a significant inverse association with urinary m^7^Gua levels (*P* for trend = 0.03). In analysis stratified by occupation, inverse correlations were observed in sedentary workers (walking < 30 min/day at work: *P* for trend = 0.06 and = 0.03 for urinary 8-OH-dG and m^7^Gua, respectively), but not in physically active workers (walking ≥ 30 min/day at work). In analysis for each intensity of leisure-time physical activity, light-intensity exercise was associated with lower levels of urinary 8-OH-dG (*P* for trend = 0.03), whereas moderate-to-high-intensity exercise was associated with lower levels of urinary m^7^Gua (*P* for trend = 0.02).

**Conclusions:**

Our results suggest that high levels of leisure-time physical activity are associated with decreased levels of DNA damage in individuals with low physical activity at work.

## Introduction

The number of people diagnosed with cancer annually is increasing globally; this trend is expected to continue in the future [1]. Several meta-analyses have reported that physical activity reduces the risk of specific types of cancer and consequent mortality [2–5]. Although the biological mechanisms underlying the cancer-preventative effects of physical activity remain unclear, DNA damage is thought to be one of the major mechanisms involved [6–8].

Useful known biomarkers of DNA damage include 8-hydroxydeoxyguanosine (8-OH-dG) and 7-methylguanine (m^7^Gua). 8-OH-dG is one of the primary oxidative stress markers associated with carcinogenesis [9], while m^7^Gua is a biomarker of DNA damage induced by methylating agents [10]. Higher levels of 8-OH-dG have been reported in patients with specific cancers including bladder, prostate, breast, and lung cancers, as well as malignant lymphomas [11, 12]. Levels of m^7^Gua are higher in colorectal tumor patients [13] and are associated with an increased risk of lung cancer [14].

Several epidemiological studies have examined the association between physical activity and 8-OH-dG levels, but their results have been inconsistent; some reported an inverse association between physical activity and 8-OH-dG [15–17], while others found no obvious association [18–20]. To the best of our knowledge, only one study examined the association between total physical activity and m^7^Gua, but failed to detect any significant associations [17]. Holtermann *et al*. reported that different domains of physical activity have different effects on health, and defined this as the “physical activity health paradox [21, 22].” Indeed, a previous study suggested that physical labor, which was investigated as a working condition, tended to have a positive correlation with 8-OH-dG [16]. Therefore, it is necessary to consider the influence of occupational physical activity when investigating the associations between leisure-time physical activity and DNA damage among workers. This cross-sectional study examined the associations between leisure-time physical activity and urinary 8-OH-dG, a DNA oxidation marker, or urinary m^7^Gua, a DNA methylation marker, with stratification by occupational physical activity.

## Materials and methods

### Participants

The participants were workers from two municipal offices in the northeastern region of Kyushu, Japan. Medical check-ups were conducted at one office (site A) in July 2009, and at the other office (site B) in November 2009, as described previously [23]. All full-time employees (*n* = 605), excluding those on long-term sick leave or maternity leave, were invited to participate in the study and asked to respond to the provided questionnaire. A total of 567 people, aged 20–68 (325 men and 242 women), agreed to participate in the study (94% response rate). Participants were excluded based on the following criteria: cancer (*n* = 13), cardiovascular disease (*n* = 11), diabetes (*n* = 8), taking anemia medication (*n* = 1), nephritis (*n* = 1), hepatitis (*n* = 3), motor function disorders (*n* = 1), musculoskeletal disorders (*n* = 3), and pregnancy (*n* = 8). We also excluded individuals whose urinary 8-OH-dG and m^7^Gua levels could not be measured owing to missing urine samples (*n* = 18), and people who had missing covariate variables (*n* = 9). Some excluded participants met two or more of these conditions. These exclusions left 501 people (294 men and 207 women), aged 20–65 years, for analysis in the study. Written informed consent was obtained from all participants. The study protocol was approved by the ethics committee of the National Center for Global Health and Medicine. This study was conducted in accordance with the Declaration of Helsinki and the Japanese Ethical Guidelines.

### Assessment of DNA damage

Urinary 8-OH-dG and m^7^Gua levels were measured as described previously [17, 24, 25]. Briefly, a urine sample was mixed with the same volume of a solution containing the ribonucleoside marker 8-hydroxyguanosine. A 20-μL aliquot of the diluted urine sample was injected into a high-performance liquid chromatography (HPLC)-1 system via the guard column. The chromatograms were recorded using a Gilson UV detector (UV/VIS-155 with 0.2 mm light path cell). Creatinine and m^7^Gua were detected at 245 and 305 nm, respectively. The 8-OH-dG fraction was collected depending on the elution position relative to the peak of the added marker, 8-hydroxyguanosine, and was automatically injected into an HPLC-2 column and detected by a Coulochem II EC detector with a guard cell and an analytical cell. The 8-OH-dG and m^7^Gua levels were adjusted against urinary creatinine levels, as creatinine is frequently used as an internal standard for normalization.

### Physical activity measurement

We evaluated the physical activity of the study participants using a self-reported questionnaire. For leisure-time physical activity, we asked participants to report their weekly leisure-time hours engaged in walking and performing light-intensity exercise (e.g., calisthenics or golf), moderate-intensity sweaty exercise (e.g., tennis or volleyball), and high-intensity exhaustion exercise (e.g., soccer or basketball). We also asked participants to report their weekly hours engaged in gardening. We assigned a metabolic equivalent (MET) value for each physical activity [26] and calculated the leisure-time physical activity (MET-hours/week) by multiplying the MET by the time (hours) for which each physical activity was performed per week (3 METs for walking and light-intensity exercise, 4 METs for moderate-intensity exercise, 8 METs for high-intensity exercise, and 3 METs for gardening). For occupational physical activity, we asked participants to report their daily time spent walking at work with six-level response options ranging from “hardly any walking (less than 10 min)” to “more than 4 hours.”

### Other variables

Serum ferritin concentrations (ng/mL) were measured by a chemiluminescence immunoassay on the Bayer ADVIA Centaur, as described previously [27]. The height and weight of each participant was measured while they were wearing light clothing with their shoes removed, and body mass index (BMI) was calculated by dividing body weight (kg) by height (m) squared. Self-reported questionnaires were used to investigate smoking status, use of antioxidant supplements, use of nonsteroidal anti-inflammatory medication, and overtime work status. Dietary habits, including coffee, green tea, vegetable, and fruit intake, during the month before the examination were evaluated using a brief self-administered diet history questionnaire (BDHQ), which was assessed for validity [28, 29].

### Statistical analysis

For participant characteristics, continuous variables were indicated by the median and interquartile range, and categorical variables were indicated by percentages. We classified the participants into three categories based on their level of leisure-time physical activity (MET-hours/week): “low” as none, “middle” as equal to or below the median of participants engaged in leisure-time physical activity (median: 8 METs-hours/week), and “high” as above the median. A log conversion of urinary 8-OH-dG and m^7^Gua levels was performed prior to analysis, as the levels did not show a normal distribution. To examine the relationship between leisure-time physical activity and urinary 8-OH-dG and m^7^Gua, we calculated the geometric means of urinary 8-OH-dG and m^7^Gua levels adjusted for age (years, continuous), sex (male or female), and workplace (site A or B) using analysis of covariance with 95% confidence intervals (CIs) according to the three categories of leisure-time physical activity (model 1). Additionally, we adjusted for BMI (kg/m^2^, continuous), smoking status (never smoker, former smoker, current smoker), coffee intake frequency (< 1 cup/day, 1 cup/day, ≥ 2 cups/day), green tea intake frequency (< 1 cup/day, 1 cup/day, ≥ 2 cups/day), vegetable intake (g/day, continuous), fruit intake (g/day, continuous), use of antioxidant supplements (yes or no), use of nonsteroidal anti-inflammatory medication (yes or no), log-transformed ferritin levels (ng/mL, continuous), overtime work (< 10 hours/month, 10–19 hours/month, ≥ 20 hours/month), and occupational physical activity (walking at work: < 10 min/day, 10–29 min/day, ≥ 30 min/day) as covariates, and obtained the geometric means of urinary 8-OH-dG and m7Gua levels (model 2). We chose these covariates based on the literature suggesting associations with 8-OH-dG and m^7^Gua levels [17, 23, 27] The trend associations were assessed using multiple linear regression analysis with the ordinal numbers 1 to 3 assigned to the lowest through highest categories of leisure-time physical activity. We also performed an analysis stratified by the level of occupational physical activity (walking at work: < 30 min/day or ≥ 30 min/day). In addition, we investigated the associations for each intensity of leisure-time physical activity (light-intensity exercise and/or walking and/or gardening, and moderate- and/or high-intensity exercise). Participants were classified into three categories: not engaged, ≤ 2 hours/week, and > 2 hours/week. We also classified participants into three groups: “none,” “light-intensity exercise (including walking and gardening),” and “moderate- and/or high-intensity exercise.” Participants who engaged in both intensities of activity (*n* = 48) were classified into the group with “moderate- and/or high-intensity exercise.” Statistical significance was set to 5% on both sides for all analyses, and SAS statistical software version 9.4 (SAS Institute Inc., Cary, NC, USA) was used for analysis.

## Results

The characteristics of the study participants according to their categories of leisure-time physical activity are shown in Table 1. Participants with higher levels of leisure-time physical activity were more likely to be male and to use antioxidant supplements, and tended to have a higher BMI; coffee, vegetable, and fruit intake; level of occupational physical activity; and serum ferritin level.

**Table 1:** Characteristics of the study participants according to leisure-time physical activity.

	Leisure-time physical activity (MET-hours/week)		
	Low	Middle	High
	(0.0)	(≤ 8.0)	(> 8.0)
*n*	252	126	123
Age (years)	40 (36–51)	44 (36–55)	44 (34–57)
Men (%)	50.8	59.5	74.0
Workplace (site A)	29.0	29.4	29.3
Body mass index (kg/m^2^)	21.8 (19.6–23.9)	22.1 (19.9–24.0)	22.6 (20.9–25.1)
Smoking			
Never smoker (%)	65.5	56.3	52.8
Former smoker (%)	10.3	17.5	21.1
Current smoker (%)	24.2	26.2	26.0
Coffee intake			
< 1 cup/day (%)	39.7	43.7	30.1
1 cup/day (%)	20.6	21.4	24.4
≥ 2 cup/day (%)	39.7	34.9	45.5
Green tea intake			
< 1 cup/day (%)	32.9	24.6	39.0
1 cup/day (%)	17.5	20.6	18.7
≥ 2 cup/day (%)	49.6	54.8	42.3
Vegetable intake (g/day)	180.3 (129.6–253.0)	183.9 (127.1–276.7)	212.9 (144.5–331.3)
Fruit intake (g/day)	44.1 (20.5–87.9)	43.9 (20.5–96.4)	73.2 (21.5–124.0)
Walking and light-intensity exercise (%)	―	69.0	61.8
Moderate-intensity exercise (%)	―	16.7	43.9
High-intensity exercise (%)	―	2.4	22.8
Gardening (%)	―	21.4	30.1
Walking at work			
< 10 min/day (%)	32.5	26.2	19.5
10–29 min/day (%)	32.5	36.5	38.2
≥ 30 min/day (%)	34.9	37.3	42.3
Overtime work			
< 10 hours/month (%)	65.5	69.8	74.8
10–19 hours/month (%)	14.3	15.1	14.6
≥ 20 hours/month (%)	20.2	15.1	10.6
Use of antioxidant supplements (%)	14.3	11.9	16.3
Use of anti-inflammatory drugs (%)	8.3	4.8	8.9
Serum ferritin (ng/mL)	85.9 (28.1–170.0)	101.1 (43.1–155.0)	115.0 (56.2–195.0)

Data are expressed as median (interquartile) or percentage.

Abbreviation: MET, metabolic equivalent.

Table 2 shows the geometric means of urinary 8-OH-dG and m^7^Gua concentrations according to leisure-time physical activity. Leisure-time physical activity tended to be inversely associated with urinary 8-OH-dG in the fully-adjusted model (model 2; *P* for trend = 0.06). The geometric means (95% CIs) of urinary 8-OH-dG levels from the lowest to highest category of leisure-time physical activity were 3.36 (3.08–3.67), 3.40 (3.07–3.76), and 3.07 (2.77–3.40) μg/g creatinine, respectively. We identified a significant inverse association between leisure-time physical activity and urinary m^7^Gua (model 2; *P* for trend = 0.03). The geometric means (95% CIs) of urinary m^7^Gua levels were 7.65 (6.94–8.44), 7.47 (6.67–8.37), and 6.83 (6.09–7.66) μg/g creatinine for the lowest to highest category of leisure-time physical activity, respectively.

**Table 2:** Geometric means and their 95% confidence intervals of urinary 8-OH-dG and m^7^Gua concentrations according to leisure-time physical activity.

	Leisure-time physical activity (MET-hours/week)			*P* for trend
	Low	Middle	High	
	(0.0)	(≤ 8.0)	(> 8.0)	
**Urinary 8-OH-dG (μg/g creatinine)**				
**All the participants (*n* = 501)**				
	(*n* = 252)	(*n* = 126)	(*n* = 123)	
Model 1	2.99 (2.82–3.18)	3.15 (2.90–3.43)	2.80 (2.57–3.05)	0.32
Model 2	3.36 (3.08–3.67)	3.40 (3.07–3.76)	3.07 (2.77–3.40)	0.06
**Stratified by occupational physical activity; *P* for interaction = 0.04**				
**Sedentary worker (walking at work < 30 min/day) (*n* = 314)**				
	(*N* = 164)	(*N* = 79)	(*N* = 71)	
Model 3	3.08 (2.73–3.48)	3.28 (2.86–3.76)	2.69 (2.34–3.09)	0.06
**Physically active worker (walking at work ≥ 30 min/day) (*n* = 187)**				
	(*n* = 88)	(*n* = 47)	(*n* = 52)	
Model 3	3.63 (3.15–4.18)	3.44 (2.92–4.07)	3.60 (3.07–4.22)	0.82
**Urinary m^7^Gua (μg/g creatinine)**				
**All the participants (*n* = 501)**				
	(*n* = 252)	(*n* = 126)	(*n* = 123)	
Model 1	6.78 (6.40–7.18)	6.57 (6.07–7.11)	6.16 (5.68–6.69)	0.06
Model 2	7.65 (6.94–8.44)	7.47 (6.67–8.37)	6.83 (6.09–7.66)	0.03
**Stratified by occupational physical activity; *P* for interaction = 0.09**				
**Sedentary worker (walking at work < 30 min/day) (*n* = 314)**				
	(*n* = 164)	(*n* = 79)	(*n* = 71)	
Model 3	7.52 (6.57–8.60)	6.80 (5.85–7.91)	6.66 (5.70–7.79)	0.03
**Physically active worker (walking at work ≥ 30 min/day) (*n* = 187)**				
	(*n* = 88)	(*n* = 47)	(*n* = 52)	
Model 3	7.83 (6.67–9.18)	8.85 (7.33–10.68)	7.35 (6.14–8.81)	0.68

Model 1: Adjusted for age (years, continuous), sex (male or female), and workplace (site A or B).

Model 2: Adjusted for model 1 covariates plus body mass index (kg/m^2^, continuous), smoking status (never smoker, former smoker, current smoker), coffee intake (< 1 cup/day, 1 cup/day, ≥ 2 cup/day), green tea intake (< 1 cup/day, 1 cup/day, ≥ 2 cup/day), vegetable intake (g/day, continuous), fruit intake (g/day, continuous), use of antioxidant supplements (yes or no), use of non-steroidal anti-inflammatory drugs (yes or no), log-transformed ferritin levels (ng/mL, continuous), overtime work (< 10 hours/month, 10–19 hours/month, ≥ 20 hours/month), and occupational physical activity (walking at work: < 10 min/day, 10–29 min/day, ≥ 30 min/day).

Model 3: Adjusted for model 2 covariates, except for occupational physical activity (walking at work: < 10 min/day, 10–29 min/day, ≥ 30 min/day).

Abbreviations: 8-OH-dG, 8-hydroxydeoxyguanosine; m^7^Gua, 7-methylguanine; MET, metabolic equivalent.

In analysis stratified by occupational physical activity (walking at walk: < 30 min/day or ≥ 30 min/day), there was a statistically significant interaction between occupational physical activity and leisure-time physical activity on urinary 8-OH-dG (*P* for interaction = 0.04). There was a suggestion of an inverse association between leisure-time physical activity and urinary 8-OH-dG in sedentary workers (walking at work < 30 min; *P* for trend = 0.06), but not in physically active workers (walking at work ≥ 30 min; *P* for trend = 0.82). Although there were no significant interactions between occupational physical activity and leisure-time physical activity on urinary m^7^Gua (*P* for interaction = 0.09), we found a significant inverse association between leisure-time physical activity and urinary m^7^Gua in sedentary workers (*P* for trend = 0.03), but not in physically active workers (*P* for trend = 0.68).

Table 3 shows the geometric means of urinary 8-OH-dG and m^7^Gua concentrations according to intensity of leisure-time physical activity. Light-intensity exercise (including walking and gardening) was associated with lower levels of urinary 8-OH-dG (*P* for trend = 0.03), whereas moderate-to-high-intensity exercise was associated with lower levels of urinary m^7^Gua (*P* for trend = 0.02).

**Table 3:** Geometric means and their 95% confidence intervals of urinary 8-OH-dG and m^7^Gua concentrations according to intensity of leisure-time physical activity.

	*n*	Urinary 8-OH-dG (μg/g creatinine)	Urinary m^7^Gua (μg/g creatinine)
**Stratification analysis**			
**Hours of leisure-time physical activity for each intensity**			
**Light-intensity exercise (including walking and gardening)** ^a^^,^ ^c^^,^ ^d^			
None	295	3.39 (3.11–3.70)	7.57 (6.87–8.34)
≤ 2 hours/week	130	3.45 (3.11–3.83)	7.33 (6.52–8.23)
> 2 hours/week	76	2.92 (2.60–3.27)	6.94 (6.11–7.88)
*P* for trend		0.03	0.14
**Moderate- and/or high-intensity exercise** ^a^^,^ ^c^^,^ ^e^			
None	410	3.31 (3.05–3.60)	7.55 (6.88–8.28)
≤ 2 hours/week	55	3.28 (2.88–3.74)	6.65 (5.75–7.68)
> 2 hours/week	36	3.10 (2.67–3.60)	6.65 (5.64–7.85)
*P* for trend		0.37	0.02
**Intensity of leisure-time physical activity** ^b^^,^ ^c^			
None	252	3.35 (3.07–3.66)	7.61 (6.90–8.39)
Light-intensity exercise (including walking and gardening)	158	3.25 (2.94–3.58)	7.44 (6.67–8.29)
Moderate- and/or high-intensity exercise	91	3.20 (2.86–3.58)	6.64 (5.85–7.52)
*P* for trend		0.28	0.02

^a^ Participants were classified into three categories for each intensity of leisure-time physical activity (light-intensity exercise and/or walking and/or gardening, and moderate- and/or high-intensity exercise): not engaged, ≤ 2 hours/week, and > 2 hours/week.

^b^ Participants were classified into three groups: “none,” “light-intensity exercise (including walking and gardening),” and “moderate- and/or high-intensity exercise.” Participants who engaged in both intensities of activity (*n* = 48) were classified into the group with “moderate- and/or high-intensity exercise.”

^c^ All analyses were adjusted for age (years, continuous), sex (male or female), workplace (site A or B), body mass index (kg/m^2^, continuous), smoking status (never smoker, former smoker, current smoker), coffee intake (< 1 cup/day, 1 cup/day, ≥ 2 cup/day), green tea intake (< 1 cup/day, 1 cup/day, ≥ 2 cup/day), vegetable intake (g/day, continuous), fruit intake (g/day, continuous), use of antioxidant supplements (yes or no), use of non-steroidal anti-inflammatory drugs (yes or no), log-transformed ferritin levels (ng/mL, continuous), overtime work (< 10 hours/month, 10–19 hours/month, ≥ 20 hours/month), and occupational physical activity (walking at work: < 10 min/day, 10–29 min/day, ≥ 30 min/day).

^d^ Additionally adjusted for moderate and high-intensity exercise (hours/week, continuous).

^e^ Additionally adjusted for light-intensity exercise, walking, and gardening (hours/week, continuous).

Abbreviations: 8-OH-dG, 8-hydroxydeoxyguanosine; m^7^Gua, 7-methylguanine.

## Discussion

This cross-sectional study examined the association between leisure-time physical activity and urinary 8-OH-dG, a DNA oxidation marker, or urinary m^7^Gua, a DNA methylation marker, in Japanese workers. We found that leisure-time physical activity showed a suggestive inverse correlation with urinary 8-OH-dG and a significant inverse association with urinary m^7^Gua, especially among sedentary workers. Further, light-intensity exercise was associated with lower levels of urinary 8-OH-dG, whereas moderate-to-high-intensity exercise was associated with lower levels of urinary m^7^Gua.

Results of previous studies on the association between physical activity and 8-OH-dG are mixed. In a study of male Japanese workers, total physical activity—including commuting, working, and sports—was inversely associated with urinary 8-OH-dG levels, independent of other lifestyle factors [17]; furthermore, moderate leisure-time exercise (less than 5 hours per week) was associated with lower concentrations of urinary 8-OH-dG [16]. In a substudy of the Japan Multi-institutional Collaborative Cohort Study, which assessed daily physical activity by using an accelerometer, showed that light-to-vigorous physical activity in women and moderate-to-vigorous physical activity in men were inversely associated with the concentration of urinary 8-OH-dG [15]. In an intervention study among patients with type 2 diabetes, aerobic exercise greatly reduced urinary 8-OH-dG levels [30]. On the other hand, the frequency of leisure-time exercise was not associated with urinary 8-OH-dG in Japanese city office workers [19, 20], and serum 8-OH-dG levels did not differ between sedentary individuals and those who exercised regularly among healthy American young adults [18]. Epidemiological evidence linking m^7^Gua to physical activity is limited. Contrary to our findings, Tamae *et al*. reported no significant associations between total physical activity (including commuting, working, and sports) and urinary m^7^Gua in healthy male Japanese workers [17].

The inconsistency of this association in different studies may be partly attributable to the different domains of physical activity assessed in each study. Physical activity in some domains may worsen DNA damage. Specifically, physical labor has shown to be positively correlated with urinary 8-OH-dG [16]. The present study found a significant interaction between leisure-time and occupational physical activity on urinary 8-OH-dG (*P* for interaction = 0.04), showing an inverse tendency of relationship between leisure-time physical activity and urinary 8-OH-dG and m^7^Gua in sedentary workers, but not in physically active workers. This finding suggests that high levels of leisure-time physical activity are associated with reduced DNA damage in sedentary workers only.

We found that light-intensity exercise was associated with lower levels of oxidative DNA-damage markers, but high-intensity exercise showed no such association. The results of a short-term trial reveal that exhausting exercise increased urinary 8-OH-dG levels [31, 32]. In contrast, mild exercise has been shown to reduce leukocyte 8-OH-dG levels in sedentary individuals [33]. These findings suggest that mild-intensity activity may decrease oxidative DNA damage, especially for among sedentary people. Regarding DNA methylation, we found that high-intensity, but not light-intensity, exercise was associated with lower levels of urinary m^7^Gua, suggesting that high-intensity, rather than light-intensity, physical activity may decrease methylated DNA damage, unlike oxidative DNA damage. Given limited data regarding the intensity of physical activity on these markers, long-term trials are required to confirm the present findings.

The molecular mechanisms underlying the association between physical activity and DNA damage are not fully understood. Oxidative DNA damage reflects reduced defense against reactive oxygen species. Among the four nucleotide bases, guanine is the most susceptible to oxidative damage. Physical activity might increase the activity of human 8-oxoguanine-DNA glycosylase (OGG1), which plays a crucial role in the base excision repair pathway, by removing 8-oxoguanine base lesions produced by reactive oxygen species [34]. Radak *et al*. reported that exercise increases the activity of OGG1 [35, 36]. Although the molecular pathway of exercise-related DNA methylation is not completely understood, recent review articles have summarized evidence demonstrating that aberrant DNA methylation is associated with physical activity [37–40]. Exercise-related DNA methylation appears to be tissue- and gene-specific [37]. In a study among gastric cancer patients, methylation of *CACNA2D3*, a known tumor-suppressor gene, was higher among those who did not exercise than among those who exercised more than an hour per week [41]. In an intervention study among breast cancer patients, aerobic exercise training for 6 months reduced methylation of *L3MBTL1*, a known tumor-suppressor gene [42].

There were several limitations to the present study. Firstly, as the design of the study was cross-sectional, it could not discuss causal relationships. Further examinations through a longitudinal study are needed. Secondly, the participants self-reported the leisure-time physical activity. Thus, bias due to inaccurate reporting cannot be ruled out. Moreover, we assessed occupational physical activity using a single question (walking time during work) and did not obtain information on domestic physical activity. Seasonal variations in physical activity and the duration of the engagement were also not considered. Thirdly, because the present study was conducted on municipal office workers, it is difficult to generalize the results of this study to individuals in other occupations. It is necessary to confirm whether similar results would be seen in groups with different characteristics.

## Conclusions

The present study provides additional evidence supporting that higher leisure-time physical activity is associated with decreased levels of DNA damage in sedentary job workers.

## Supporting information

S1 Table Questionnaire in Japanese. (DOCX)

S2 Table Questionnaire in english. (DOCX)
