# Supplementary material for: Leisure-time physical activity and DNA damage among Japanese workers
Source: PLoS One. 2019 Feb 15;14(2):e0212499. doi: 10.1371/journal.pone.0212499 (PMC6377137; doi:10.1371/journal.pone.0212499)
Supplement: S1 Table — (DOCX) [file pone.0212499.s001.docx]

**健　康　調　査　票**

あてはまる番号に○をつけるか、数字を記入ください。

- 先月、残業をどのくらいしましたか。実際に行った時間をお答えください。

| 1) しなかった 2) 10時間未満 3) 10～19時間 4) 20～29時間 5) 30～39時間 6) 40時間以上 |
| --- |

- 仕事中、平均して1日どのくらい歩きますか。

| 1) ほとんど歩かない（10分未満） 2) 10～29分 3) 30～59分 4) 1時間～2時間未満 5) 2時間～4時間未満 6) 4時間以上 |
| --- |

- 余暇や休憩時間に、週1回以上、運動しますか（散歩を含む）。

| 1) する 2) しない　または　するが週1回未満 |
| --- |

**↓**

| 散歩・ウォーキング（通勤は除く） 週に［　　　.　　］時間 |
| --- |
| その他の軽いスポーツ 例　体操・ゴルフ 週に［　　　.　　］時間 |
| 中程度のスポーツ（息がはずむ） 例　テニス・バレーボール 週に［　　　.　　］時間 |
| 激しいスポーツ（息が上がる） 例　サッカー・バスケットボール 週に［　　　.　　］時間 |

- 週1回以上、庭や田畑で作業をしますか。

| 1) する　**→**　週に［　　　.　　］時間 2) しない　または　週1回未満 |
| --- |

- たばこを吸いますか。

| 1) 吸わない 2) やめた 3) 吸う |
| --- |

- 週1回以上の頻度で、ビタミンやミネラルのサプリメントをとっていますか。

| 1) とっていない 2) とっている　**→**　どのようなサプリメントですか。 |
| --- |

商品名［　　　　　　　　　　　　　　　］　　会社名［　　　　　　　　　　　　　　　］

商品名［　　　　　　　　　　　　　　　］　　会社名［　　　　　　　　　　　　　　　］

- 週1回以上の頻度で、痛みや炎症を抑える薬を飲んでいますか（ステロイド剤は除く）。

| 1) 飲んでいない 2) 飲んでいる |
| --- |

その他、食事調査の内容については下記URL参照

簡易型自記式食事歴法質問票BDHQ（<http://www.ebnjapan.org/>）
