# Supplementary material for: Leisure-time physical activity and DNA damage among Japanese workers
Source: PLoS One. 2019 Feb 15;14(2):e0212499. doi: 10.1371/journal.pone.0212499 (PMC6377137; doi:10.1371/journal.pone.0212499)
Supplement: S2 Table — (DOCX) [file pone.0212499.s002.docx]

**Health-Related Behavior Questionnaire**

For each question, circle the number for your response and fill in any corresponding blanks when appropriate.

- How much overtime did you work last month? Please indict the actual amount of time.

| 1) None 2) Less than 10 hours 3) 10‒19 hours 4) 20‒29 hours 5) 30‒39 hours 6) More than 40 hours |
| --- |

- While you are doing your job every day, how much do you walk, on average?

| 1) Hardly at all (less than 10 minutes) 2) 10‒29 minutes 3) 30‒59 minutes  4) 1 to less than 2 hours 5) 2 to less than 4 hours 6) 4 or more hours |
| --- |

- Do you exercise (including taking walks) one or more times a week during your leisure time or during breaks?

| 1) Yes 2) No/Less than once a week |
| --- |

**↓**

| Time spent walking (other than for commuting) [__.__] hour(s)/week |
| --- |
| Time spent doing other low-intensity exercise (e.g., calisthenics, golf)  [__.__] hour(s)/week |
| Time spent doing moderate-intensity exercise (that makes you sweat and increases your breathing rate; e.g., playing tennis, volleyball) [__.__] hour(s)/week |
| Time spent doing high-intensity exercise (that makes you breathless and brings you to the point of exhaustion; e.g., playing soccer, basketball) [__.__] hour(s)/week |

- Do you work in a garden or field one or more times a week?

| 1) Yes. → [__.__] hour(s)/week 2) No/Less than once a week |
| --- |

- Do you smoke?

| 1) No 2) No, but I used to. 3) Yes |
| --- |

- Do you take vitamins or mineral supplements one or more times a week?

| 1) No 2) Yes **→** What kinds? |
| --- |

Name［　　　　　　　　　　　　　　　］　　Manufacturer［　　　　　　　　　　　　　　　］

- Do you take drugs (other than steroids) to control pain or inflammation one or more times a week?

| 1) No 2) Yes |
| --- |

- Dietary assessment
  *Reference: brief-type self-administered diet history questionnaire, BDHQ
  (http://www.ebnjapan.org/)
